# Development and Implementation of a High-Throughput Compound Screening Assay for Targeting Disrupted ER Calcium Homeostasis in Alzheimer's Disease

**DOI:** 10.1371/journal.pone.0080645

**Published:** 2013-11-15

**Authors:** Kamran Honarnejad, Alexander Daschner, Armin Giese, Andrea Zall, Boris Schmidt, Aleksandra Szybinska, Jacek Kuznicki, Jochen Herms

**Affiliations:** 1 Department of Translational Brain Research, DZNE – German Center for Neurodegenerative Diseases, Munich, Germany; 2 Center for Neuropathology and Prion Research; Ludwig Maximilian University, Munich, Germany; 3 Graduate School of Systemic Neurosciences, Ludwig Maximilian University, Munich, Germany; 4 Clemens Schöpf Institute of Chemistry and Biochemistry, Technische Universität Darmstadt, Darmstadt, Germany; 5 Laboratory of Neurodegeneration, International Institute of Molecular and Cell Biology, Warsaw, Poland; 6 Munich Cluster for Systems Neurology (SyNergy), Munich, Germany; Sungkyunkwan University, Republic of Korea

## Abstract

Disrupted intracellular calcium homeostasis is believed to occur early in the cascade of events leading to Alzheimer's disease (AD) pathology. Particularly familial AD mutations linked to Presenilins result in exaggerated agonist-evoked calcium release from endoplasmic reticulum (ER). Here we report the development of a fully automated high-throughput calcium imaging assay utilizing a genetically-encoded FRET-based calcium indicator at single cell resolution for compound screening. The established high-throughput screening assay offers several advantages over conventional high-throughput calcium imaging technologies. We employed this assay for drug discovery in AD by screening compound libraries consisting of over 20,000 small molecules followed by structure-activity-relationship analysis. This led to the identification of Bepridil, a calcium channel antagonist drug in addition to four further lead structures capable of normalizing the potentiated FAD-PS1-induced calcium release from ER. Interestingly, it has recently been reported that Bepridil can reduce Aβ production by lowering BACE1 activity. Indeed, we also detected lowered Aβ, increased sAPPα and decreased sAPPβ fragment levels upon Bepridil treatment. The latter findings suggest that Bepridil may provide a multifactorial therapeutic modality for AD by simultaneously addressing multiple aspects of the disease.

## Introduction

Alzheimer's disease (AD) is the most common form of dementia [1]. Major breakthroughs in understanding the underlying mechanisms that cause AD within the last few decades have not yet yielded effective disease-modifying therapies. The major hallmarks of AD are the accumulation of intracellular neurofibrillary tangles of hyperphosphorylated tau protein and extracellular plaques of β-amyloid (Aβ) protein in brain [1]. Current AD drug development mainly focuses on targeting these two major pathological features. However, there is evidence that preceding the manifestation of those hallmarks and cognitive deficits, the neuronal calcium homeostasis is disturbed as a result of aging or due to missense mutations in Presenilin genes – the most common cause of early onset familial AD (FAD) [2]–[7]. Long-term disruption of calcium homeostasis has been shown to both trigger and accelerate Aβ and Tau pathologies [8]–[12]. Moreover, calcium dysregulation as an early event in AD progression plays a key role in synaptic failure and neuron loss [13], [14]. Notably, the latter irreversible pathological events correlate best with the severity of dementia [14], [15]. Calcium alterations in peripheral tissues have been even proposed as diagnostic markers for mild AD [16], [17]. Interestingly, Memantine, one of the only few approved drugs for treatment of moderate-to-severe AD patients, is an NMDA receptor antagonist, which by inhibition of sustained calcium influx leads to stabilization of intracellular calcium homeostasis [18]. Therefore, restoring disrupted calcium homeostasis as an early event leading to cellular dysfunction may open novel avenues to more efficient treatment of AD patients. Hence, we examined the possibility of stabilizing intracellular store calcium homeostasis, particularly in the endoplasmic reticulum (ER), as an innovative target for AD drug discovery. To that end, we developed a high throughput compound screening assay and screened over 20,000 small molecules which led to the identification of lead structures which can reverse the familial Alzheimer's disease-linked mutant Presenilin 1 (FAD-PS1) mediated disruption of ER calcium homeostasis.

## Materials and Methods

### Cell culture and cell lines

Human embryonic kidney 293 (HEK293) cells were cultured in Dulbecco's modified eagle medium (DMEM) supplemented with 10% fetal bovine serum and 1% penicillin/streptomycin while being incubated at 37°C, 5% CO_2_ and 90% humidity. Stable PS1 lines (generously provided by Dr. S. Lammich) were carrying PS1 variants that were cloned into pcDNA3.1/Zeo(+) and selected via Zeocin antibiotic resistance [19]. The PS1 lines were then stably transfected with YC3.6/pcDNA3 construct (kindly provided by Dr. A. Miyawaki) and respectively isolated by G418 antibiotic resistance leading to generation of double stable lines [20]. The APP-, C99- and APPsw/PS1-M146L-overexpressing HEK293 lines were kindly provided by Dr. S. Lichtenthaler and Dr. H. Steiner and cultured as it has been previously described [21], [22].

### Compound Library

DIVERSet™ 1 and 2 libraries (ChemBridge Corp., San Diego, CA, USA), each containing a diverse collection of 10,000 hand-synthesized small molecules (in total 20,000 compounds) as well as a medium size ion channel ligand library (Enzo Life Sciences GmbH, Lörrach, Germany) comprising 72 further structures were used for high throughput compound screening. Compounds fulfilled the “Lipinski's rule of 5”, indicating their high druglikeness [23].

### High throughput calcium imaging assay and automated image analysis

For the primary screen, HEK293 cells stably expressing PS1-M146L and Yellow Cameleon 3.6. (YC3.6) were seeded at 13,000 cells/well in 40 µl of growth medium on collagen-coated 384-well CellCarrier plates (PerkinElmer, Rodgau, Germany). After 6 h, using an automated pipetting robot (Bravo; Agilent Technologies, Santa Clara, CA, USA), library compounds were added to each well at the final concentration of 10 µM in 1% DMSO, each in 4 replicates. All plates contained Thapsigargin (TP; 1 µM; Calbiochem, Darmstadt, Germany), Cyclopiazonic acid (CPA; 20 µM; Calbiochem) and 3,4,5-trimethoxybenzoic acid 8-(diethylamino)octyl ester (TMB-8; 50 µM; Sigma-Aldrich, Taufkirchen, Germany) as positive controls, as well as untreated and DMSO vehicle-treated wells. After 24 h using the pipetting robot, DRAQ5 (Biostatus Ltd, Leicestershire, UK), a far-red fluorescent nuclear dye, was added to each well at the final concentration of 500 nM. After 2 h, plates were measured for Carbachol (CCh)-induced calcium release using the Opera® high-throughput confocal imaging platform (PerkinElmer Cellular Technologies GmbH, Hamburg, Germany). Throughout imaging of the entire plate, 37°C temperature, 5% CO_2_ and 90% humidity was maintained in the plate chamber. Using a 442 nm laser, YC3.6 was excited and its CFP and YFP emissions were separated respectively using 483/35 nm and 540/75 nm filters. Additionally, using a 640 nm laser DRAQ5 dye was excited and its emission was collected by 690/50 nm filter in order to locate the nuclei. Imaging was performed with a 20× water immersion autofocus objective. The duration of the entire time-lapse calcium imaging for each well was 23.5 s. This was achieved by imaging at 2.5 s interval resolution prior to dispensing CCh (for 5 s) to monitor the basal calcium levels. Next, the CCh-induced calcium rise and decay was monitored for 18.5 s post dispensing. Imaging was performed first at 1 s interval resolution immediately after dispensing (for 5 s) and subsequently at 2.5 s interval resolution (for 12.5 s). During dispensing, 10 µl of CCh (Calbiochem) diluted in HBSS (10 µM) was injected to each well concurrent with calcium imaging by an automated dispensing unit which is part of the Opera® platform. Imaging was performed sequentially for all 384 wells. Using Acapella® software (PerkinElmer Cellular Technologies GmbH), an automated image analysis tool was developed to convert fluorescent signals to numerical values. Here, DRAQ5 and YC3.6 signals were used respectively to segment single cell nuclei and single cell boundaries. After assigning each cell to its segmented nuclei and excluding the cells positioned at the edges of the imaging frames, calcium transients for every cell were monitored by plotting the kinetics of YFP/CFP versus time and finally normalizing the signals using the equation, ΔF/F_0_  =  (F − F_0_)/F_0_, where F is the measured fluorescence signal at any given time and F_0_ is the average fluorescence signal of the time points preceding CCh application. The peak amplitude of calcium rise upon CCh injection was the primary output of automated image analysis at single cell level. Non-responsive cells to CCh were excluded from analysis by setting an arbitrarily defined threshold. The average peak amplitude of all responsive cells in each well was calculated as the final readout in this assay.

### Data mining

Data mining, clustering and identification of the lead structures were performed with the Benchware DataMiner software (Tripos, St. Louis, MO, USA).

### Cytotoxicity assay

The cytotoxicity of the compounds was assessed *in vitro* using the 3-(4,5-dimethylthiazol-2-yl)-2,5-diphenyltetrazolium bromide (MTT) cell proliferation assay kit (Roche Diagnostics GmbH, Mannheim, Germany) according to manufacturer's instructions and previously described protocols [24]. In brief, HEK293 cells were cultured at a density of 35,000 cells/well in 96-well cell culture plates (Nunc GmbH, Langenselbold, Germany). On the next day, compounds (10 µM) were added to separate wells in triplicates. Cells viability was analyzed after 24 h incubation with the compounds. For this purpose, 10 µl of MTT (5 mg/ml in PBS) was pipetted to each well, followed by 4 h incubation at 37°C. The formed formazan crystals were dissolved by adding 100 µl of 10% SDS (dissolved in 0.01 M HCl) to each well and the plates were shaken vigorously to ensure complete solubilization. The absorbance was measured at 560 nm using a microtiter-plate reader (FLUOstar Optima, BMG Labtech GmbH, Ortenberg, Germany). Values are presented as percentage of viable cells.

### Aβ measurements

Levels of three different Aβ species (Aβ38, Aβ40 and Aβ42) were measured using sandwich ELISA. Pools of HEK293 cells stably transfected with APPsw/PS1-M146L or APP were used to study the effect of compounds on Aβ generation. According to Page et. al. [22], cells were seeded at a density of 200,000 cells/well in collagen/poly-L-lysine (PLL)-coated 24-well plates and incubated for 24 h in growth medium. Next, the medium was exchanged with 500 µl of fresh medium containing either Bepridil (3–30 µM, Sigma-Aldrich) or the positive controls DAPT (10 µM, Calbiochem) and Sulindac sulfide (50 µM, Sigma-Aldrich) or vehicle. After 16 h conditioned medium was collected and the levels of secreted Aβ38, Aβ40 and Aβ42 fragments were quantified using “Human (6E10) Abeta 3-Plex” sandwich ELISA immunoassay kit (Meso Scale Discovery, Rockville, MD, USA) according to the instructions of the manufacturer. In brief, 150 µl of blocker reagent was added to each well of the ELISA plate and incubated for 1 h at room temperature, followed by 3× washing using TRIS wash buffer. Next, 25 µl of detection antibody was added to each well. At appropriate dilution, each of the samples or calibration standards were added to separate wells of ELISA plate and incubated for 2 h at room temperature, followed by 3× washing using TRIS wash buffer. Finally, 150 µl of read buffer was added to the wells. The light emission after electrochemical stimulation was measured using Sector Imager 2400 reader (Meso Scale Discovery). Based on the values generated with calibration standards, corresponding concentrations of Aβ species were calculated using the Meso Scale Discovery Workbench software. All measurements were performed in four replicates.

### sAPPα and sAPPβ measurements

Levels of sAPPα and sAPPβ fragments were measured using sandwich ELISA in a similar fashion as in Aβ measurements, in either HEK293 cells stably expressing APP or APPsw/PS1-M146L. Using the collected conditioned medium, the levels of secreted sAPPα and sAPPβ fragments were quantified using sAPPα/sAPPβ sandwich ELISA immunoassay kit (Meso Scale Discovery) according to the instructions of the manufacturer.

### Statistical data analysis

GraphPad Prism 5.0b (GraphPad Software, San Diego, CA, USA) was used for statistical analysis of the data. Values represent mean ± standard deviation. To test significance, two-tailed student's t-test was performed and differences were considered statistically significant if p<0.05. The Z′-factors were computed using the equation Z′  =  1 − [(3σ_TP_ + σ_DMSO_)/|μ_TP_ − μ_DMSO_|] according to Zhang et al. [25], where TP was used as a positive and DMSO as a negative control (μ: mean; σ: standard deviation).

## Results

### FAD-PS1 mutations enhance the amplitude of CCh-induced calcium release and the number of responsive cells

**Figure 1:**
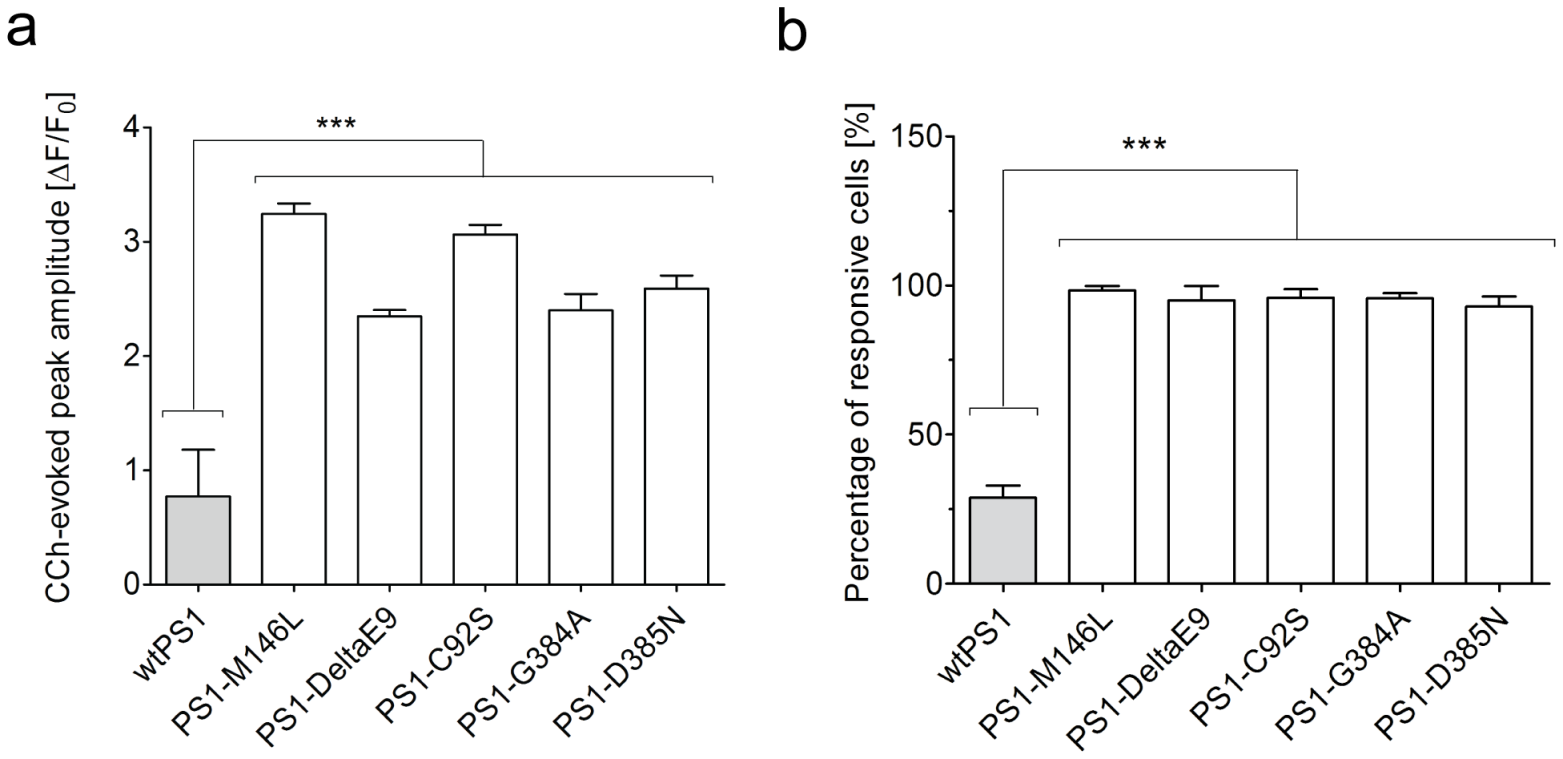
CCh–induced calcium release in HEK293 carrying PS1 mutations. (**a**) The average peak amplitude of CCh-induced calcium release is significantly potentiated in FAD and inactive PS1 mutants compared to wild type PS1 cells (*** P<0.001). (**b**) The average number of responsive cells to CCh is remarkably increased in cells expressing FAD and inactive PS1 mutants compared to wild type PS1 cells (*** P<0.001).

Mutations in presenilins (PS1 and PS2) account for the vast majority of early onset familial Alzheimer's disease (FAD) cases. These mutations result in enhancement of inositol 1,4,5-trisphosphate (IP_3_) receptor sensitivity [26], [27]. As expected, the peak response of CCh-evoked calcium release in FAD-PS1 lines was approximately 3 folds higher than in wild type PS1 line (Figure 1a). Moreover, a remarkable increase in the number of CCh-responsive cells was consistently detected in all FAD-PS1 lines. In wild type PS1 line, only 29% of the cell population was CCh-responsive, whereas in all FAD-PS1 mutant lines, over 95% of the cell population responded to CCh (10 µM) (Figure 1b). Taken together, FAD-PS1 expression enhances the number of responsive cells to CCh and amplifies the peak response of CCh-evoked calcium response. Likewise, the expression of a γ-secretase-deficient mutant form of PS1 (PS1-D385N) results in increased responsiveness to CCh and augmented calcium release from ER upon CCh stimulation (Figure 1a and 1b). In the next set of experiments, the augmented CCh-evoked calcium release in FAD-PS1 expressing cells was used as the target to screen for compounds that can reverse exaggerated calcium release towards physiological levels.

### High throughput compound screening assay enables the discovery novel lead structures

We addressed intracellular store calcium dyshomeostasis as an innovative target for drug discovery with a novel FRET single-cell-based calcium imaging technique in a fully automated high-throughput kinetic assay on the “Opera” system (PerkinElmer) for compound screening.

**Figure 2:**
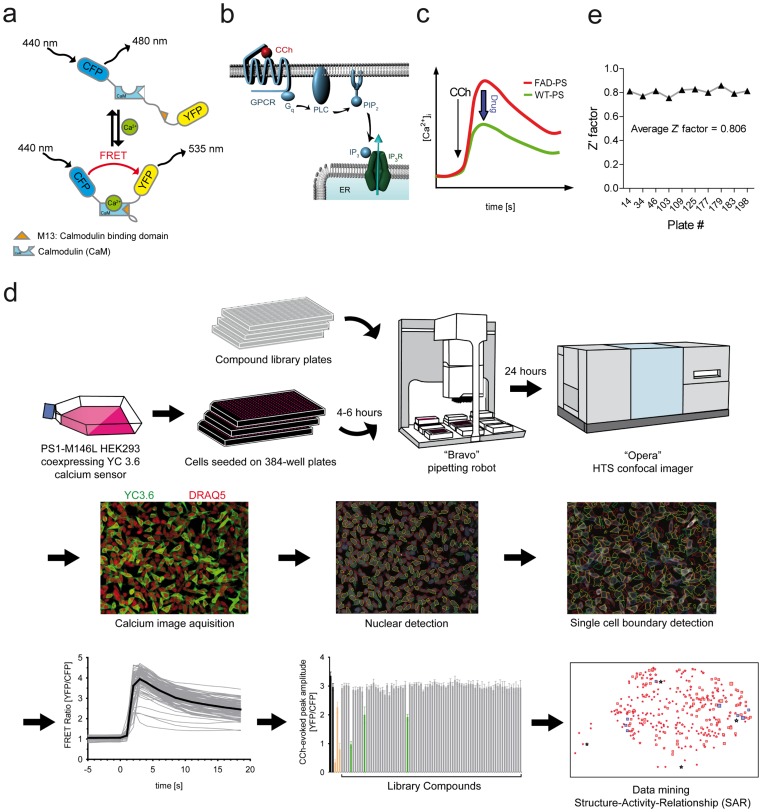
Workflow of the high-throughput FRET calcium imaging based compound screening assay and data analysis. (**a**) Structure of the calcium sensor YC3.6 that is a fusion protein composed of CFP and YFP attached via calmodulin (CaM) and a CaM-binding peptide (M13). Calcium binding brings CFP and YFP together, shifting the emission of 480 nm to 535 nm upon excitation at 440 nm. (**b**) CCh application initiates a pathway, which results in calcium release from ER. CCh exposure leads to G-coupled activation of PLC catalyzing the hydrolysis of the membrane-associated PIP_2_ molecule to IP_3_. Binding of IP_3_ molecule to IP_3_ receptor channels (IP_3_R) on the ER membrane in turn leads to opening of IP_3_R channels and calcium release from ER to cytosol. (**c**) Representative calcium transients of CCh-evoked calcium release in cells expressing FAD-linked PS mutant versus wild type PS. FAD-PS expressing cells exhibit an exaggerated calcium release upon CCh exposure. The arrow shows the time point at which CCh is applied. The HTS screening rationale was to identify drugs that can restore the FAD-PS-associated potentiation of CCh-evoked calcium release to the level of wild type PS. (**d**) HEK293 cells stably expressing PS1-M146L and YC3.6 calcium indicator are seeded in 384-well format plates. 6–8 h post seeding, using a pipetting robot, library compounds are added to separate wells. After 24 h, to stain nuclei, DRAQ5 is added to each well using the pipetting robot. After 2 h, plates are confocally imaged by “Opera” system, which is equipped with a fast dispensing unit applying CCh to each well during time-lapse imaging. An image analysis tool within the “Acapella” software is developed to automatically analyze single cell calcium transients. Using DRAQ5 nuclear segmentation, image analysis tool detects the boundaries of individual cells in the first time point and measures then the intensities of in FRET–acceptor and –donor over the course of imaging. The FRET efficiency of individual cells are then calculated and normalized. For each cell, the signal maximum (peak) is determined. The compounds which attenuated the peak amplitude of CCh-induced calcium release to <90% of the DMSO controls were regarded as hit. Finally, by data mining and determining the structure-activity-relationships (SAR) of the entire library consisting of over 20,000 compounds, active lead structures were identified. (**e**) Z′-factor as a measure for the robustness of the screening assay is evaluated for ten randomly selected imaged plates. The average Z′-factor for the screened plates exceeded 0.8.

Yellow Cameleon 3.6 (YC3.6), a superior genetically-encoded FRET-based calcium probe with expanded dynamic range and fast kinetics [20], was introduced to HEK293 cells as a tool to monitor both the basal calcium concentrations and the released calcium from ER in real-time by confocal imaging. YC3.6 is composed of CFP and YFP domains linked via calmodulin (CaM) and a CaM-binding peptide (M13). Upon calcium binding it undergoes a conformational change and thereby FRET efficiency increases (Figure 2a) [20]. The assay readout was the peak response of potentiated inositol-1,4,5-trisphosphate receptor (IP_3_R)-evoked calcium release from ER in HEK293 cells carrying a disease-causing mutated form of PS1 (PS1-M146L). Agonist-induced IP_3_ production by CCh results in calcium release from ER (Figure 2b). As presented here and reported by others as well [28], FAD-PS mutations mediate exaggerated CCh-induced calcium release compared to wild type PS1 expressing cells (Figures 1a and 2c). Representative traces of CFP, YFP and their FRET ratio in response to 10 µM of two different agonists (CCh and Histamine) are presented in Figure S1. Notably, CCh-evoked calcium responses were evaluated at several different CCh concentrations in PS1-M146L cells, from which the EC_50_ for CCh was found to be at 162 nM (Figure S2a). The most remarkable difference in the peak response of CCh-evoked calcium release in FAD-PS1 versus wild type PS1 lines was detected at 10 µM CCh (Figure S2f). The latter stands right before the upper plateau of dose response is reached, thus offering a wide dynamic range for identification of compounds that can decrease the ER calcium response in PS1-M146L cells (Figure S2a).

As illustrated in Figure 2d, HEK293 cells stably coexpressing FAD-linked PS1-M146L mutation and YC3.6 were seeded on 384-well optical bottom plates. After 6 h, compounds from the library plates were added to separate wells using a pipetting robot. After 24 h incubation with compounds, DRAQ5 nuclear marker was added to the wells. After 2 h, time-lapse calcium imaging was performed and CCh-induced calcium release was monitored sequentially for each well with the use of YC3.6 calcium indicator. In addition, the signal of DRAQ5 dye was also collected throughout the entire course of time-lapse imaging. Using “Acapella” software (PerkinElmer), an automated image analysis tool was developed to convert fluorescent signals in large number of cell populations to numerical values. To that end, DRAQ5 signal was used to detect all nuclei in each frame and the YC3.6 signal was used to assign the detected single cell boundaries to their corresponding segmented nuclei over the entire course of time-lapse calcium imaging. On average, approximately 150–200 cells were detected for each well. For every detected cell, calcium transients were measured by calculating YFP/CFP over the course of imaging. The peak response amplitude of the calcium rise upon CCh injection was the output of automated analysis at single cell level. The ability to simultaneously monitor calcium transients for all individual cells of a well enabled us to filter out CCh-non-responsive cells from the analysis by setting an arbitrarily defined threshold. The average peak amplitude of all responsive cells in a well was calculated as the final output of the image analysis tool.

The performance of the high-throughput compound screening assay remained very robust throughout screening of 201 plates (Z′-factor >0.8). Z′-factors for ten randomly selected screened plates are presented in Figure 2e. The average Z′-factor for those ten plates was determined to be 0.806±0.029, reflecting the robustness of the assay for high-throughput screening (HTS).

**Figure 3:**
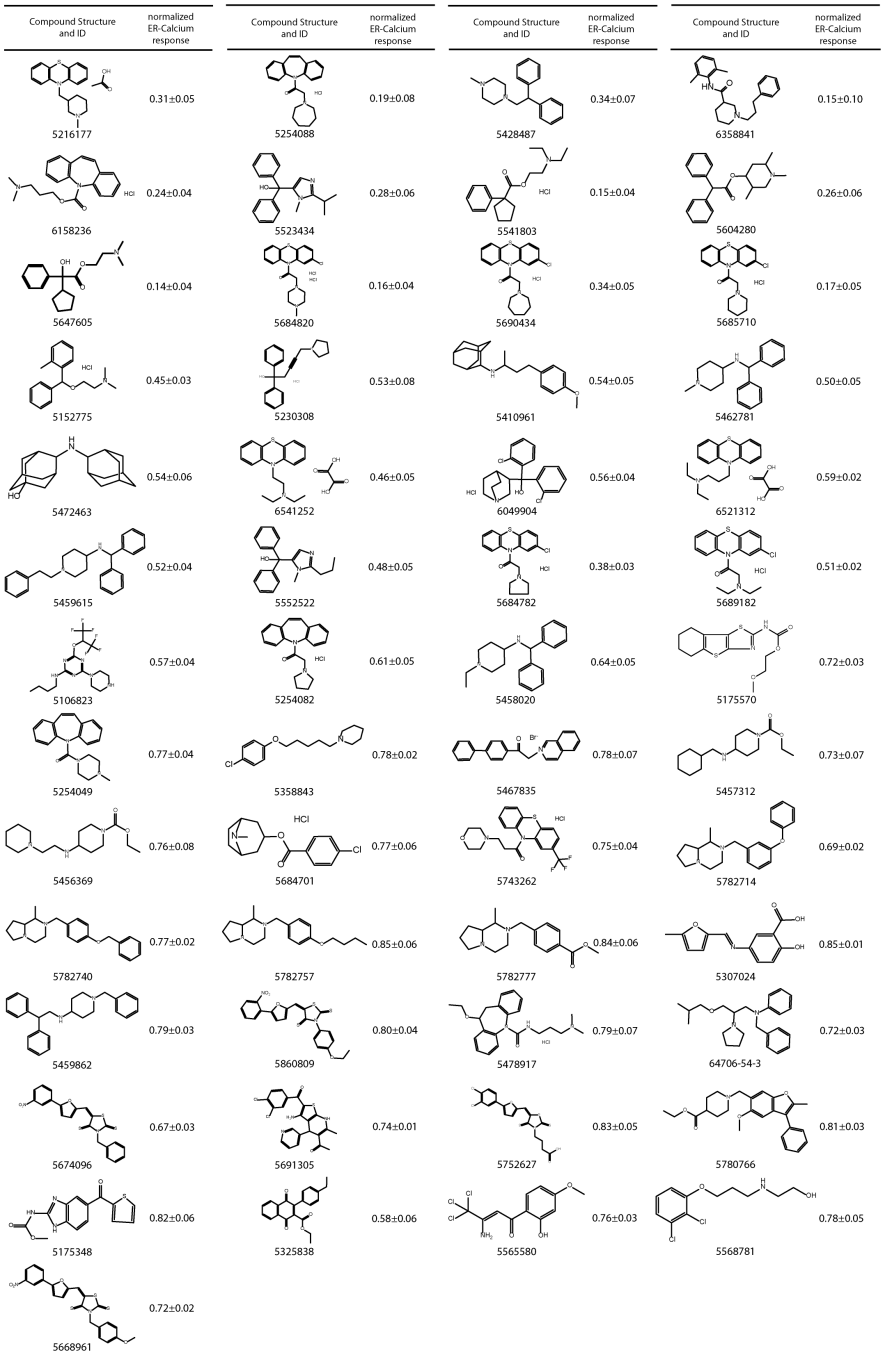
Active structures identified from primary HTS screen. Shown are 53 small molecules identified from the primary screen with their chemical structure and the corresponding normalized mean ER calcium response ± standard deviation values generated at 10 µM, as a measure for their activity.

After filtering the autofluorescent and highly toxic compounds, 53 active small molecule hits were identified from the primary screen (Figure 3). A compound was regarded as active, if the peak response of calcium release in cells treated with that compound was 90% or smaller than the peak response of DMSO-treated controls on the same plate. To each library compound a score typically <1.0 was assigned indicating a measure for its efficacy, calculated by dividing the peak response of calcium release in cells treated with that given compound to the peak response of DMSO-treated controls on the same plate. Hereafter, we refer to this value as “normalized ER calcium response”. In Figure 3, the list of all hits from the primary screen including their chemical structures and corresponding average normalized ER calcium response values are presented.

**Figure 4:**
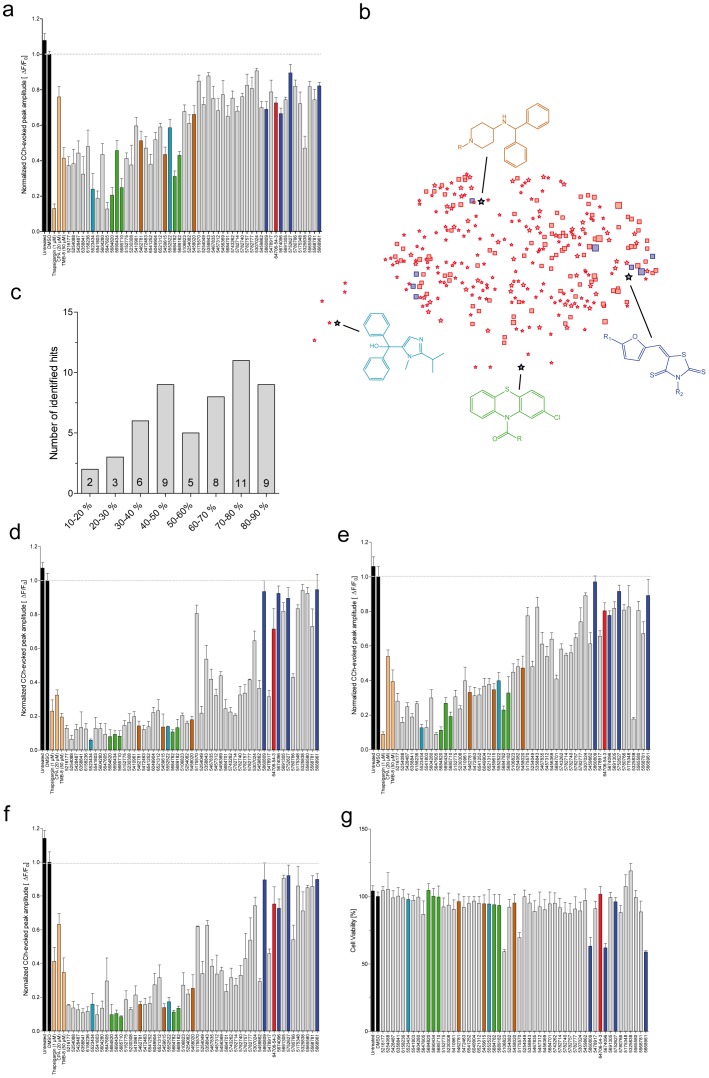
Validation of primary hits, preliminary structure-activity-relationship (SAR) analysis and their *in vitro* cytotoxicity. (**a**) The activity of all 53 primary hits was validated in PS1-M146L/YC3.6 line. All the hits were capable of reducing the peak response of CCh-induced calcium release to <90% of DMSO-treated controls. (**b**) The structure-activity-relationship (SAR) map of the screened compounds. The symbols represent compound clusters generated by Benchware DataMiner software. The distance between the clusters correlates with the similarity between their chemical structures. The number of compounds within a cluster is illustrated by the size of the symbol. A cluster with more than 50% active compounds is represented by a star, and marked in blue if the actual number of active compounds is greater than four. The highlighted identified lead structures belong to compound classes Thiazolidine (blue), Phenothiazine (green), Imidazole (turquoise) and Benzhydrilpiperidinamine (brown). Primary hits were also active in HEK293 cells expressing (**e**) PS1-DeltaE9/YC3.6, (**f**) PS1-C92S/YC3.6, and (**g**) PS1-D385N/YC3.6, by attenuating the mutant PS1-induced amplified calcium release. (**c**) and (**d**) The hits from the primary screen were classified into 8 categories based on their efficacy in lowering the CCh-evoked calcium release. These categories are separated according to the value of normalized ER calcium response. The noted numbers in each category indicates the number of compounds belonging to that category. (**h**) Viability of HEK293 cells treated with the primary screen hits was assessed by means of MTT assay after 24 h compound treatment. Values are presented as percentage of viable cells. In (a), (e), (f) and (g), the peak response of DMSO-treated control is set to one. Each color denotes a different lead structure in (a), (b), (e), (f), (g) and (h). The data for analogous molecules belonging to the same lead structure are marked with the same color.

The activity of the entire set of 53 hits in terms of reducing the peak response of CCh-evoked calcium release was validated and confirmed in PS1-M146L line (Figure 4a). Moreover, all primary hits were capable to attenuate the CCh-evoked calcium release in three other cell lines expressing either different FAD-PS1 mutations (PS1-DeltaE9 and PS1-C92S) or a γ-secretase-deficient PS1 mutant (PS1-D385N) (Figures 4d–4f). This indicates that the activity of the identified hits in normalizing the exaggerated FAD-PS1-mediated CCh-evoked calcium release is indeed not only specific to the FAD-PS1 mutation used in the primary screen, but present across other examined PS1 mutations as well.

53 structures identified from the primary screen were classified into different categories based on their efficacy in attenuating the peak response of the CCh-evoked calcium release (Figure 4c). These categories are separated according to the corresponding values of normalized ER calcium response, presented as percentages in relation to the peak response of DMSO-treated control.

Next, we performed an MTT assay in order to assess the cytotoxicity of the entire set of 53 hits generated from the primary screen. Majority of identified active compounds showed no toxicity and HEK293 cells treated with 10 µM of the compounds for 24 h remained viable (Figure 4g). Treatment with only 5 compounds, 3 of which belonging to Thiazolidine lead structure, resulted in 30–40% reduction in cellular viability.

After preliminary structure-activity-relationship (SAR) assessments with the entire collection of library compounds using “Benchware DataMiner” software (Tripos), 4 different lead structures were identified. Those structures belonged to following compound classes: Thiazolidine, Phenothiazine, Imidazole and Benzhydrilpiperidinamine (Figure 4b, S3, S4, S5 and S6). To that end, first the library consisting of 20,000 compounds was imported to the data mining software and active compounds (normalized ER calcium response <0.9) were selected. Using the “OptiSim” algorithm groups of similar compounds, called clusters, were identified. Subsequently inactive compounds were added to the existing clusters. Then the clusters were combined according to their structures in order to reduce their number. Then a preliminary SAR map of the clusters was generated and clusters with more than 50% active compounds were represented as stars and clusters with less than 50% active compounds as rectangles. In addition, all clusters with more than 4 active compounds were colored in blue and otherwise in red. The sizes of the symbols correlate directly with the number of compounds in each cluster (Figure 4b).

### Effect of Bepridil on CCh-evoked calcium release from ER

**Figure 5:**
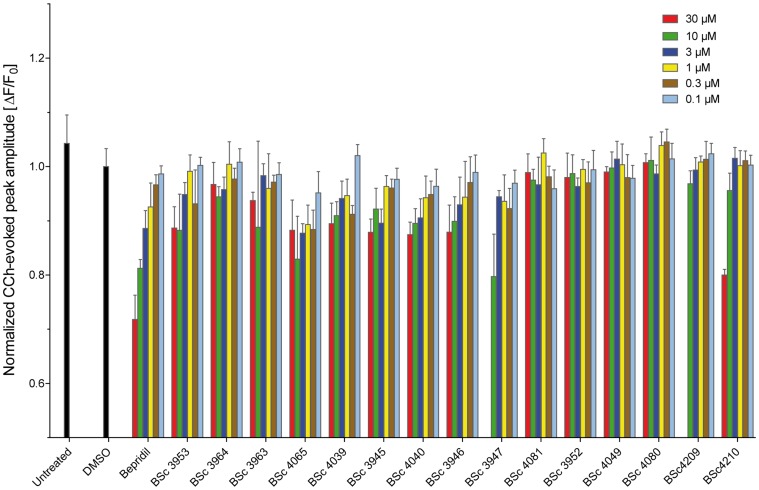
Dose-dependent effect of Bepridil and its derivative structures on the amplitude of CCh-evoked ER calcium release in PS1-M146L cells. The effect of Bepridil and its 15 synthesized derivative structures were tested at 30, 10, 3, 1, 0.3 and 0.1 µM. The peak response of DMSO-treated control is set to one. The relative peak amplitude of CCh-evoked calcium release is plotted for each treatment condition. Compounds BSc3947 and BSc4209 were toxic at 30 µM concentration.

In addition to 4 discovered lead structures, the HTS led to the identification of Bepridil, a calcium antagonist drug that was previously shown to be beneficial against AD through simultaneously targeting β- and γ-secretases [21]. In view of the detected Bepridil activity in dampening the exaggerated FAD-PS1-mediated calcium release and the promising indications linking it to lowered Aβ generation, we synthesized 15 derivative structures in an attempt to generate Bepridil-analogous molecules with improved efficacy (Figure S7). The derivatisation strategy aimed at exploring the contribution of different fragments to the potency and efficacy by removal of these moieties (BSc4040 and BSc4209). We varied the lipophilicity (BSc4040 and BSc3946), solubility, basicity (BSc4049) and membrane permeation by introduction of an ammonium salt (BSc3947) or sulfonic acid (BSc3963) and carboxylic acids (BSc3964 and BSc4065). Using the same HTS assay employed in primary screening, we measured dose-dependent effects for Bepridil and its synthesized derivatives at 30, 10, 3, 1, 0.3 and 0.1 µM (Figure 5). The chemical modifications in the structure of Bepridil did not further improve the efficacy in attenuating the CCh-evoked calcium release (Figure 5). Therefore, in the following experiments the original Bepridil structure identified from the primary screen was further characterized to determine its mode of action. Notably, Bepridil had no effect on the peak response of CCh-evoked calcium release in wild type PS1 expressing cells (Figure S8a). However, at all CCh concentrations tested, pretreatment with Bepridil led to decreased peak amplitude of calcium release from ER in PS1-M146L cells, as it was also the case with Thapsigargin (Figure S2a–c). In addition, the time course of Bepridil incubation shows that the decrease in CCh-evoked calcium response is already present after 30 minutes treatment, which does not seem to further reduce within the period of 48 hours investigated (Figure S8b). Moreover, the dose-dependent effects of Thapsigargin as well as an identified antagonist hit (compound 5647605) on the peak amplitude of CCh-evoked calcium release (10 µM), are respectively presented in Figures S2d and S2e.

### Effect of Bepridil on APP processing and Aβ generation

**Figure 6:**
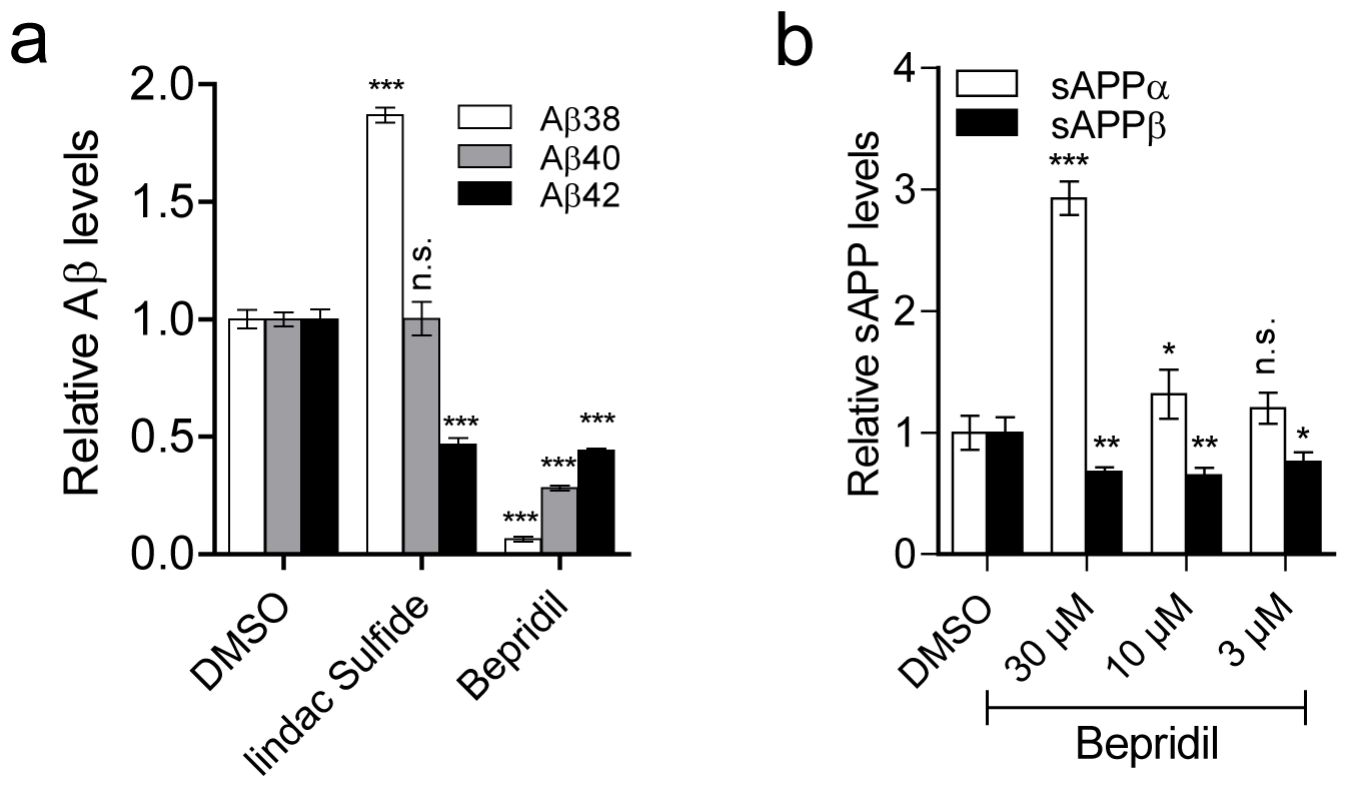
Effect of on Bepridil on APP processing. (**a**) Reduced production of Aβ38, Aβ40 and Aβ42 after 16 h Bepridil (30 µM) treatment in HEK293 cells coexpressing APPsw and PS1-M146L. Sulindac sulfide (50 µM) was used as a γ-secretase modulator control. (**b**) Increased levels of sAPPα and decreased sAPPβ secreted fragments after 16 h treatment with Bepridil in HEK293 cells coexpressing APPsw and PS1-M146L. (n.s.: non-significant; * P<0.05, ** P<0.01 and *** P<0.001).

Mitterreiter and colleagues have shown that Bepridil treatment decreases the formation of Aβ by shifting the balance of APP processing from amyloidogenic β-cleavage towards non-amyloidogenic α-cleavage [21]. In line with their observation, we also detected reductions in the level of secreted Aβ38, Aβ40 and Aβ42 peptides upon 16 h exposure of APPsw/PS1-M146L-overexpressing HEK293 cells with Bepridil at 30 µM (Figure 6a). Likewise, in APP-overexpressing HEK293 cells we detected lower Aβ38, Aβ40 and Aβ42 generation after Bepridil treatment (30 µM) for 16 h (Figure S9a). However, upon Bepridil treatment at lower concentrations (10 µM and 3 µM), we detected reduced Aβ38 and Aβ40, but increased Aβ42 levels (Figure S9a). Treatment of C99-overexpressing HEK293 cells with Bepridil (30 µM) also leads to decreased Aβ38 and Aβ40 and increased Aβ42 levels, suggesting an inverse γ-secretase modulator (iGSM), as previously also described [21] (Figure S9b). The reduction in the Aβ amounts was accompanied by an increase in sAPPα and a decrease in sAPPβ secreted fragments in a dose-dependent manner, indicating that Bepridil treatment indeed enhances the activity of α-secretase and inhibits the activity of β-secretase in two different cell lines (Figure 6b and S9c). Furthermore, we also tested the effect the other active compounds derived from the primary calcium screen on Aβ peptide production. Depending on the compound tested, we detected increased, decreased or unchanged Aβ levels upon 16 h exposure of APPsw/PS1-M146L HEK293 cells with the compounds (Figure S10).

## Discussion

Here we describe the development and implementation of a high-throughput compound screening assay targeting ER calcium dysregulation as an innovative approach for AD drug discovery. As opposed to the majority of AD drug discovery strategies that target late stage disease hallmarks, this approach targets one of the earliest and most upstream events in AD progression before the appearance of characteristic AD pathological features. Targeting late events in the course of the AD, during which the disease has likely reached an irreversible stage, could be one of the reasons for the consistent recent failure of disease-modifying AD drug candidates targeting Aβ and tangle pathologies in late clinical phases [29]. To the best of our knowledge, the possibility of targeting disrupted ER store calcium homeostasis as an upstream event in disease pathogenesis has never been examined in AD drug discovery in the past.

The HTS assay developed offers several advantages compared to current calcium measurement screening technologies. Firstly, the use of genetically-encoded calcium sensors as opposed to conventional synthetic calcium-sensitive organic dyes allows monitoring long-term intracellular calcium dynamics without the drawbacks caused by dye toxicity, loading, washing and leakage. In addition to being able to follow long-term calcium dynamics, short-term calcium imaging can be performed at multiple time points, which could even be spread over several days. Since the “Opera” HTS platform is equipped with an environmental control chamber, excellent long-term cell viability conditions are ensured by maintaining regulated temperature, humidity and CO_2_. Secondly, the developed HTS assay allows performing rapid automated dispensing of reagent jets to individual wells during calcium measurements with no time lag between dispensing and imaging. The latter is ideal for kinetic measurements that require rapid imaging with no delay post dispensing, e.g. fast agonist-induced calcium release. Thirdly, the single-cell-based nature of this assay in combination with automated image analysis enables the detection of even slight changes in calcium levels which cannot be achieved with the use of conventional single-well-based calcium measurement screening technologies (e.g. FLIPR) [30]. Moreover, the ability to simultaneously monitor calcium transients for individual cells of a well, allows applying multiple filtering parameters during image analysis to set apart different cell subpopulations from each other, e.g. “active” from “inactive”, “responsive” from “non-responsive”, “transfected” from “untransfeced” cells, etc. The latter is not possible in single-well-based calcium measurement assays. Furthermore, the assay offers competitive robustness reflected by Z′-factor >0.8. Overall, the aforementioned advantages of the developed HTS assay enabled us to identify drugs, which by having even modest effects on exaggerated IP_3_R-evoked calcium signals, may be beneficial for AD therapy.

Calcium alterations associated with FAD-PS expression provide ideal means to investigate the disruption of ER calcium homeostasis. FAD-PS-dependent calcium alterations in intracellular calcium stores have been linked to synaptic dysfunction, the underlying basis of cognitive impairment in AD [31]. Nevertheless, the potential of the developed HTS assay is not only restricted to FAD drug discovery. Early studies indicate that the disrupted ER calcium release correlates with Aβ and Tau pathologies in AD [32]. During physiological aging [2], [33] and in many neurodegenerative diseases [7], [34] the neuronal store calcium homeostasis becomes impaired. However, the alterations in ER calcium homeostasis during aging are much more subtle [35]. Notably, age is the main risk factor for developing sporadic AD [36]. Moreover, PS1 mutations are also associated with heart failure and cardiac diseases because of similar alterations in ER calcium signaling as in AD [37]. Therefore, targeting intracellular store calcium homeostasis in HTS assays may allow the identification of drugs relevant for treatment of undesired effects associated with physiological aging and a wide range of neurodegenerative and cardiac diseases. As recently reviewed by Chadwick et al., ER is not a classical AD drug target, however due to its multifactorial involvement in several cellular aspects of AD, even modest modulation in its function may present tremendous therapeutic efficacy [38].

Screening of over 20,000 small molecules using the HTS assay yielded the discovery of four lead structures and 53 primary hit molecules, one of them being Bepridil, a calcium channel blocker. Bepridil specifically attenuated the FAD-PS1-mediated exaggerated ER calcium release, without affecting the latter in wild type PS1 expressing cells. Time course of Bepridil incubation showed that already after 30 minutes, Bepridil can lower the amplitude of CCh-evoked calcium release without further decreasing it within the next 48 hours in FAD-PS1 cells. The latter indicates that Bepridil-dependent decrease in calcium release from ER is unlikely to be the consequence of ER calcium depletion, caused by for example long term blockade of voltage-gated calcium channels. Bepridil is reported to modulate APP processing by simultaneously affecting the activity of β- and γ-secretases [21]. Hence, we synthesized 15 Bepridil derivatives in an attempt to identify analogous structures with improved efficacy and potency in restoring the exaggerated IP_3_R-evoked calcium release in cells carrying FAD-linked PS1 mutations. However, these preliminary SAR assessments indicated that the modifications in the chemical structure of Bepridil do not further improve the activity of synthesized derivatives in the ER calcium release assay.

Alterations in intracellular calcium homeostasis can directly affect Aβ production [9]. Indeed, many of the active compounds identified from the primary calcium screen either increased or decreased Aβ generation. Such a wide range of effects on Aβ generation is rather predictable, since most likely those compounds target different components of intracellular calcium homeostasis, thus, also differently affecting APP processing. In accordance with the findings of Mitterreiter et al. [21], we also detected less Aβ generation upon Bepridil treatment in cells overexpressing C99, which is the β-cleaved product of APP and the substrate for γ-secretase. Treatment with Bepridil decreased Aβ38 and Aβ40 levels, but on the other hand, increased Aβ42 amounts. Mitterreiter et al. have also described such a concurrent iGSM feature for Bepridil. This might also explain our observation that in APP-overexpressing cells, Bepridil treatment at lower concentrations (10 µM and 3 µM) increases, but at higher concentration (30 µM) decreases Aβ42 levels. However, Aβ38 and Aβ40 levels were decreased at all Bepridil concentrations in all cell types tested. In other words, it appears that at low Bepridil concentrations, the iGSM effect of Bepridil (increase in Aβ42 levels) overbalances the reduced BACE1 activity (decrease in Aβ42 levels), whereas at higher Bepridil concentrations this effect is vice versa.

Mitterreiter et al. have demonstrated that Bepridil can inhibit β-secretase cleavage of APP by mildly raising the membrane-proximal endosomal pH, while independently modulating γ-secretase activity as well [21]. Moreover, evidence suggests that calcium ions can directly regulate the activity of γ-secretase [39] and BACE1 [40]. Therefore, stabilization of ER calcium homeostasis by Bepridil may present an alternative mode of action which could result in reduced activity of γ-secretase and BACE1, thus leading to lowered Aβ production. Mitterreiter et al. report that APP protein expression level is indeed unaffected by Bepridil treatment [21]. Therefore the possibility that reduced APP protein levels may account for Bepridil-dependent Aβ reduction can be excluded.

Novel molecular target(s) of Bepridil are yet to be determined. However, based on the known function of Bepridil as a calcium channel blocker, one may speculate that by dampening the hyperactivated calcium channels located on the ER [27], [41], Bepridil could stabilize the disturbed ER calcium homeostasis. Indeed, treatment with ryanodine receptor (RyR) blocker dantrolene was shown to reduce Aβ burden, increase PSD-95 expression and improve learning and memory in a APPsw-expressing mouse model of AD [42]. Moreover, PS holoprotein has been shown to form passive leak calcium channel on the ER membrane, affecting ER calcium homeostasis [19], [43]. Therefore, future studies are necessary to closely examine whether Bepridil exerts any modulatory effect on the activity of ER calcium receptor channels or the passive calcium leakage through PS holoprotein.

In this report, we focused on characterization of Bepridil, a hit identified from the HTS. However, other lead structures and hits identified from the primary screen may also provide therapeutic potential for AD treatment, which shall be investigated in future.

## Supporting Information

Figure S1 **FRET donor, acceptor and efficiency response traces to carbachol and histamine.** **(a)** FRET donor (CFP), **(b)** FRET acceptor (YFP), **(c)** raw FRET, and **(d)** normalized FRET efficiency response traces to 10 µM Carbachol agonist. Similarly, lower panels represent **(e)** FRET donor (CFP), **(e)** FRET acceptor (YFP), **(f)** raw FRET, and **(g)** normalized FRET efficiency response traces to 10 µM Histamine agonist.
(TIF)

Figure S2 **Dose-dependent response of agonists and antagonists on the peak amplitude of calcium response in PS1-M146L HEK293 cells.** Dose response effect of Carbachol agonist on the peak amplitude of calcium release in **(a)** untreated, **(b)** Thapsigargin-treated (500 nM) and **(c)** Bepridil-treated (10 µM) cells. Dose-dependent effects of antagonists **(d)** Thapsigargin, and **(e)** the hit molecule 5647605, on the peak amplitude of CCh-evoked calcium release (10 µM). **(f)** Calcium peak response for three doses of CCh in PS1-M146L versus wtPS1 expressing cells.
(TIF)

Figure S3 **Phenothiazine lead structure.** Shown are the 11 compounds belonging to the lead structure Phenothiazine. Their chemical structure, physical properties and mean normalized CCh-evoked calcium release peak response ± standard deviation are presented at 10 µM as a measure for their activity in the ER calcium release assay.
(TIF)

Figure S4 **Thiazolidine lead structure.** Shown are the 7 compounds belonging to the lead structure Thiazolidine. Their chemical structure, physical properties and normalized CCh-evoked calcium release peak response are presented at 10 µM as a measure for their activity in the ER calcium release assay.
(TIF)

Figure S5 **Benzhydrilpiperidinamine lead structure.** Shown are the 5 compounds belonging to the lead structure Benzhydrilpiperidinamine. Their chemical structure, physical properties and mean normalized CCh-evoked calcium release peak response ± standard deviation are presented at 10 µM as a measure for their activity in the ER calcium release assay.
(TIF)

Figure S6 **Imidazole lead structure.** Shown are the 3 compounds belonging to the lead structure Imidazole. Their chemical structure, physical properties and mean normalized CCh-evoked calcium release peak response ± standard deviation are presented at 10 µM as a measure for their activity in the ER calcium release assay.
(TIF)

Figure S7 **Bepridil lead structure.** Shown are Bepridil and 15 synthesized derivatives, their chemical structure, physical properties and the mean normalized CCh-evoked calcium release peak response± standard deviation at 10 µM as a measure for their activity in the ER calcium release assay.
(TIF)

Figure S8 **Characterization of the effect of Bepridil on the amplitude of CCh-evoked ER calcium release.** **(a)** Bepridil (30 µM) does not alter the amplitude of CCh-evoked ER calcium release in wildtype PS1-expressing HEK293 cells. The peak response of DMSO-treated control is set to one. Thapsigargin (1 µM), CPA (20 µM) and TMB-8 (50 µM) were used as positive controls. **(b)** The time course of Bepridil (10 µM) incubation effect on the amplitude of normalized CCh-evoked ER calcium release in HEK293 cells expressing PS1-M146L. (n.s.: non-significant and *** P<0.001).
(TIF)

Figure S9 **Effect of on Bepridil on APP processing.** **(a)** Altered production of Aβ38, Aβ40 and Aβ42 after 16 h treatment of APP-overexpressing HEK293 cells with Bepridil. DAPT (10 µM), a γ-secretase inhibitor, was used as a positive control. **(b)** Altered production of Aβ38, Aβ40 and Aβ42 after 16 h treatment of C99-overexpressing HEK293 cells with Bepridil (30 µM). Sulindac sulfide (50 µM), a γ-secretase modulator, was used as a positive control. **(c)** Increased levels of sAPPα and decreased sAPPβ secreted fragments after 16 h treatment with Bepridil in APP-overexpressing HEK293 cells. (n.s.: non-significant; * P<0.05, ** P<0.01 and *** P<0.001).
(TIF)

Figure S10 **Effects of the active compounds from the calcium HTS on Aβ production.** Altered production of Aβ38, Aβ40 and Aβ42 after 16 h treatment of HEK293 cells coexpressing APPsw and PS1-M146L with the active structures identified from the calcium HTS. DAPT (10 µM) was used as a γ-secretase inhibitor control.
(TIF)
