# Supplementary figures and images for: Development and Implementation of a High-Throughput Compound Screening Assay for Targeting Disrupted ER Calcium Homeostasis in Alzheimer's Disease
Source: PLoS One. 2013 Nov 15;8(11):e80645. doi: 10.1371/journal.pone.0080645 (PMC3829862; doi:10.1371/journal.pone.0080645)

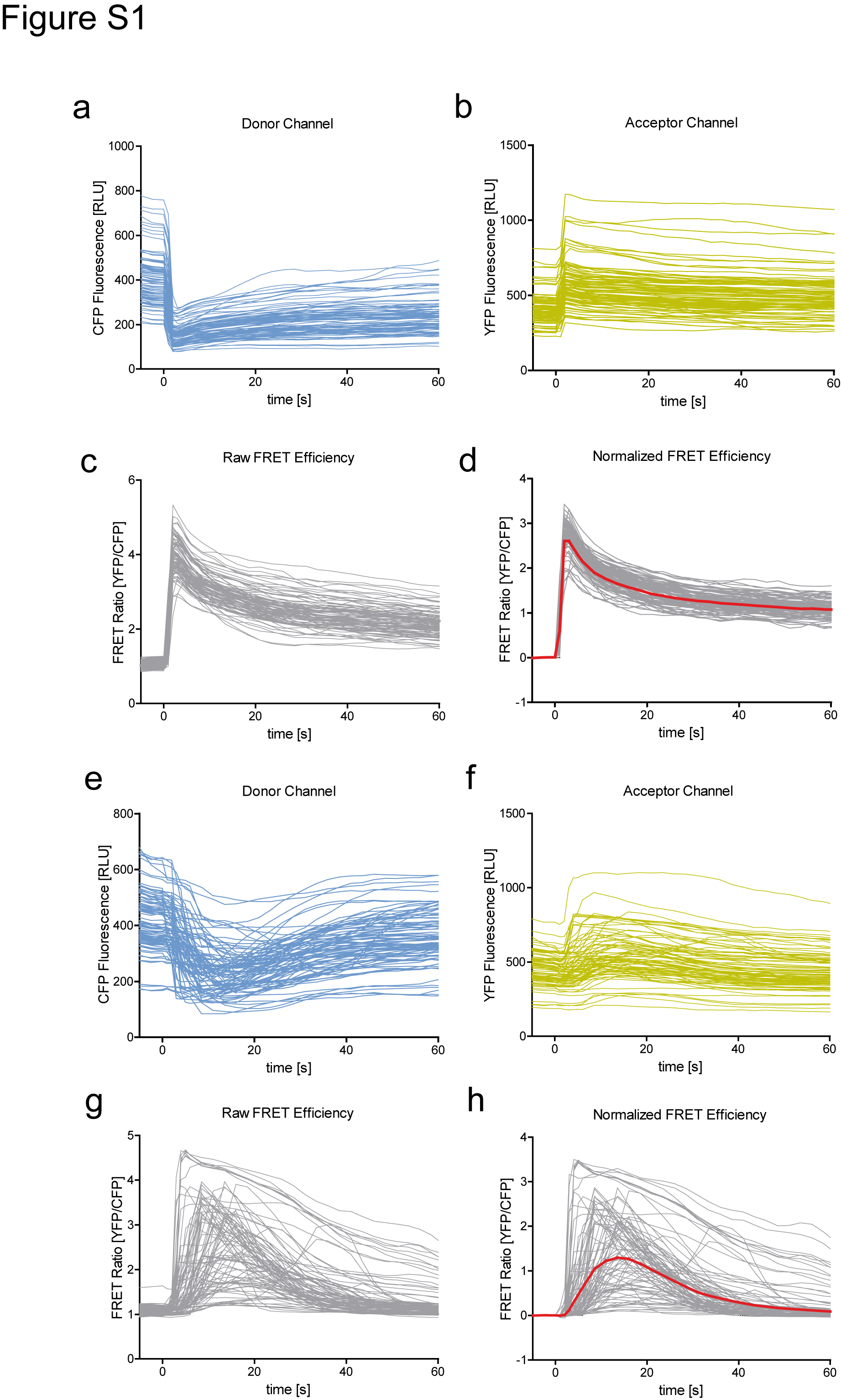

Supplement: Figure S1 — FRET donor, acceptor and efficiency response traces to carbachol and histamine. (a) FRET donor (CFP), (b) FRET acceptor (YFP), (c) raw FRET, and (d) normalized FRET efficiency response traces to 10 µM Carbachol agonist. Similarly, lower panels represent (e) FRET donor (CFP), (e) FRET acceptor (YFP), (f) raw FRET, and (g) normalized FRET efficiency response traces to 10 µM Histamine agonist. (TIF) [file pone.0080645.s001.tif]

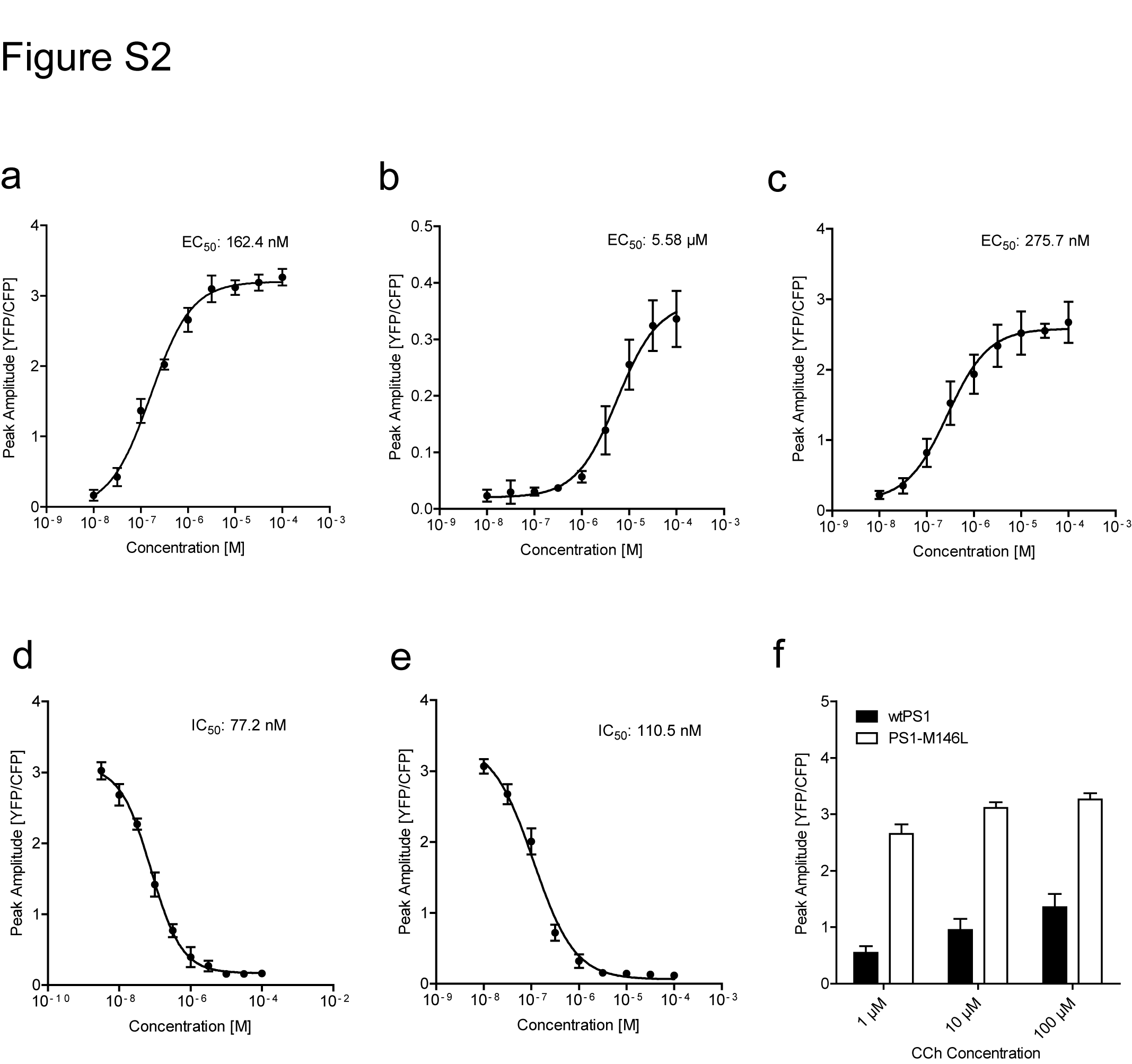

Supplement: Figure S2 — Dose-dependent response of agonists and antagonists on the peak amplitude of calcium response in PS1-M146L HEK293 cells. Dose response effect of Carbachol agonist on the peak amplitude of calcium release in (a) untreated, (b) Thapsigargin-treated (500 nM) and (c) Bepridil-treated (10 µM) cells. Dose-dependent effects of antagonists (d) Thapsigargin, and (e) the hit molecule 5647605, on the peak amplitude of CCh-evoked calcium release (10 µM). (f) Calcium peak response for three doses of CCh in PS1-M146L versus wtPS1 expressing cells. (TIF) [file pone.0080645.s002.tif]

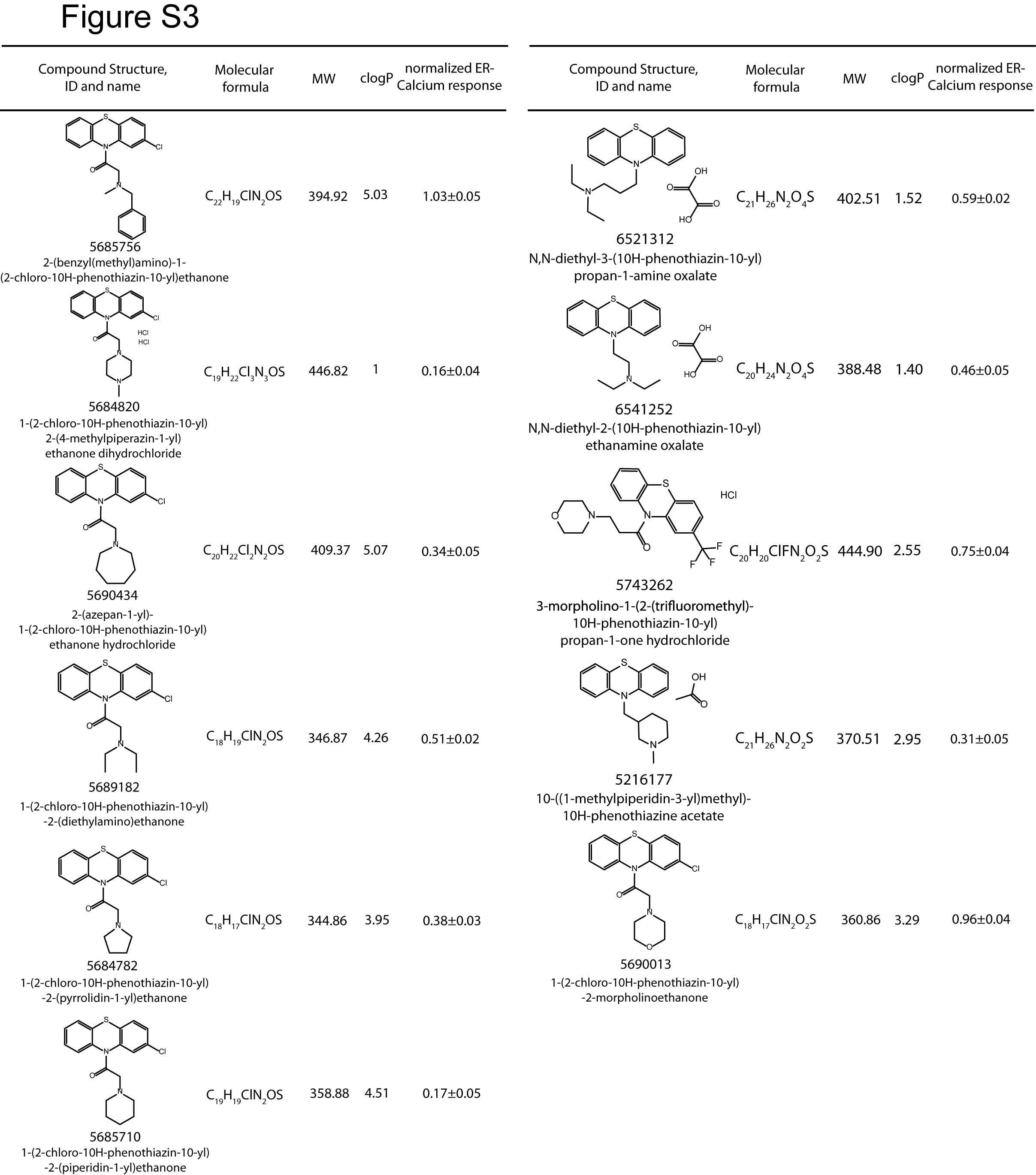

Supplement: Figure S3 — Phenothiazine lead structure. Shown are the 11 compounds belonging to the lead structure Phenothiazine. Their chemical structure, physical properties and mean normalized CCh-evoked calcium release peak response ± standard deviation are presented at 10 µM as a measure for their activity in the ER calcium release assay. (TIF) [file pone.0080645.s003.tif]

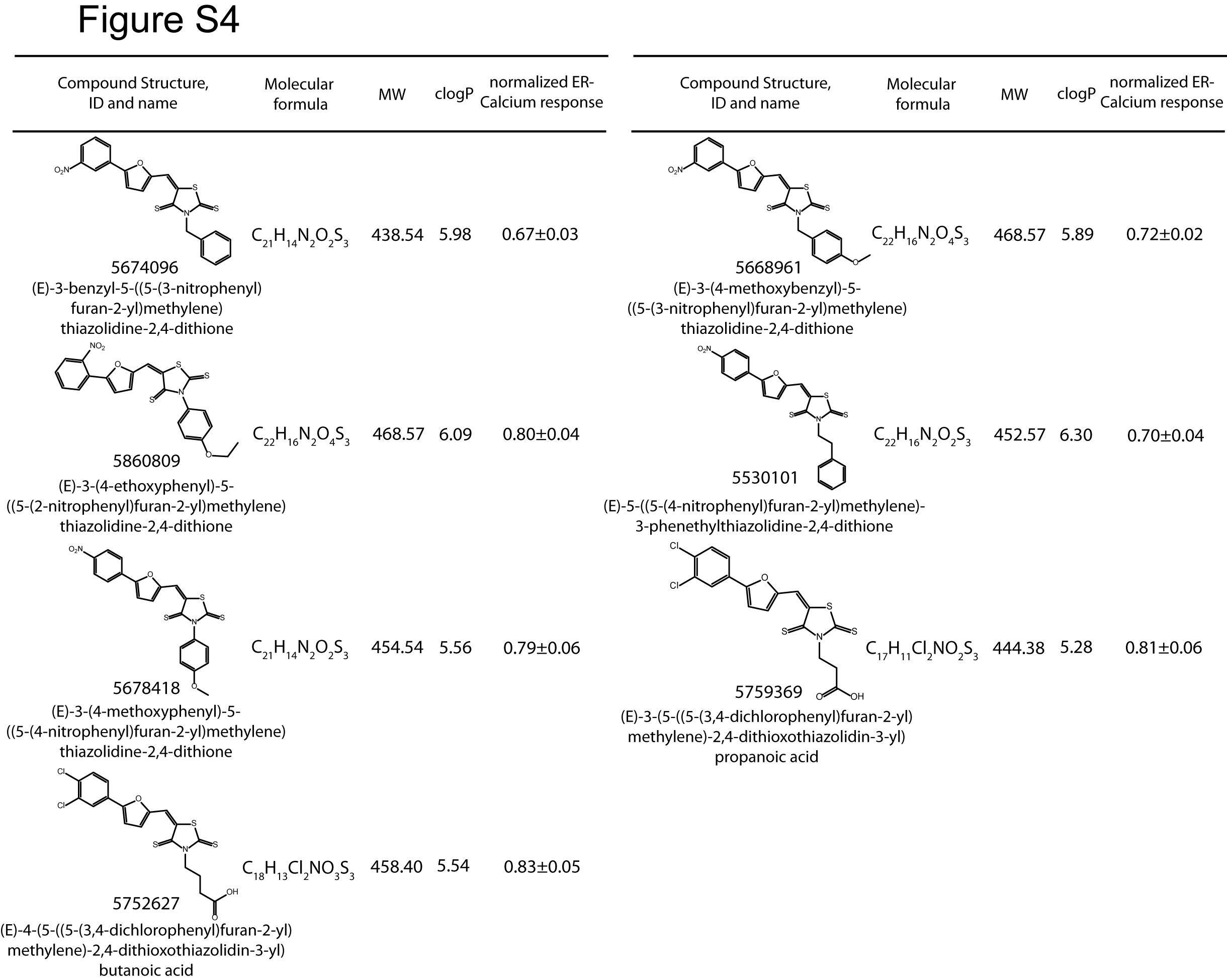

Supplement: Figure S4 — Thiazolidine lead structure. Shown are the 7 compounds belonging to the lead structure Thiazolidine. Their chemical structure, physical properties and normalized CCh-evoked calcium release peak response are presented at 10 µM as a measure for their activity in the ER calcium release assay. (TIF) [file pone.0080645.s004.tif]

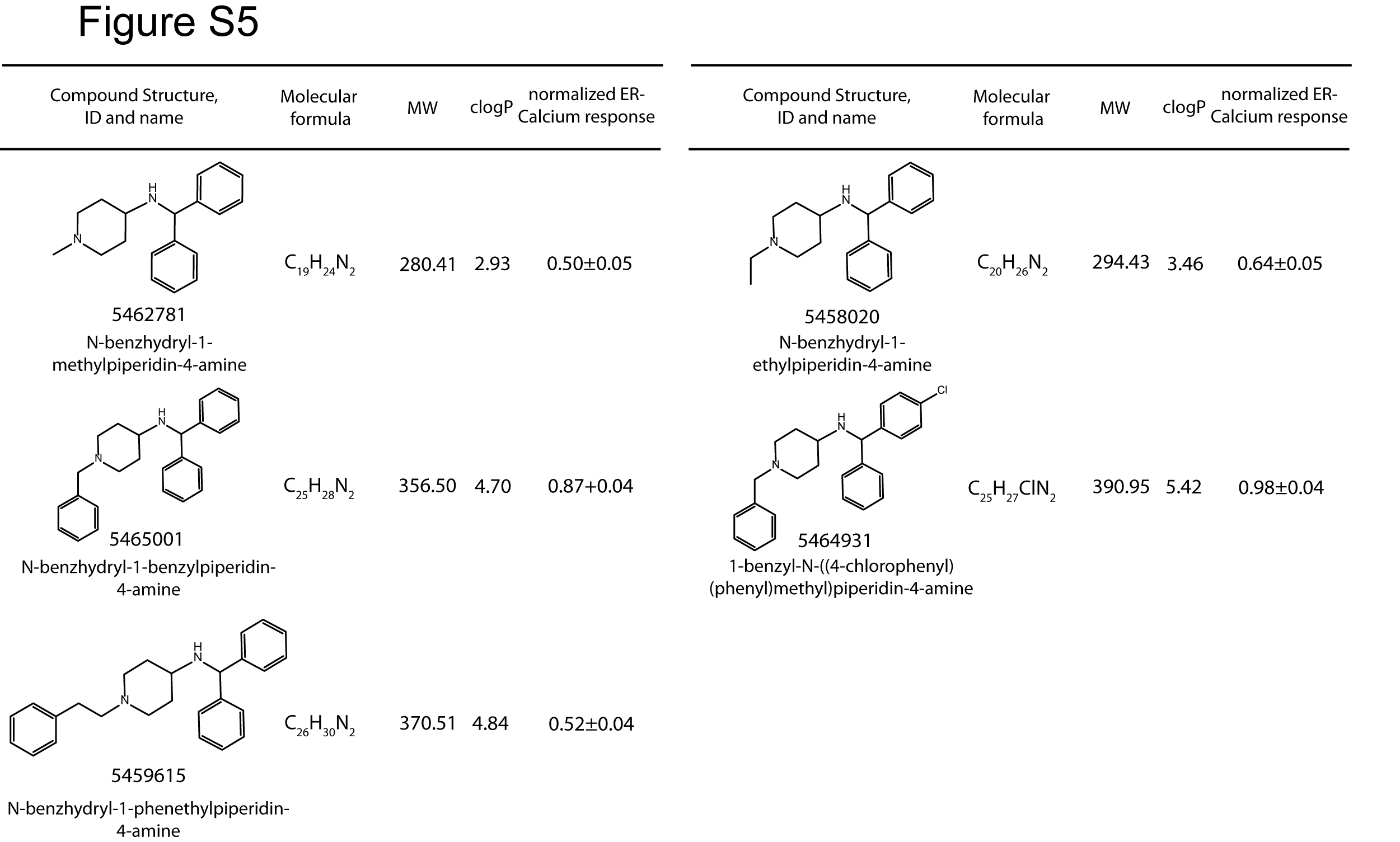

Supplement: Figure S5 — Benzhydrilpiperidinamine lead structure. Shown are the 5 compounds belonging to the lead structure Benzhydrilpiperidinamine. Their chemical structure, physical properties and mean normalized CCh-evoked calcium release peak response ± standard deviation are presented at 10 µM as a measure for their activity in the ER calcium release assay. (TIF) [file pone.0080645.s005.tif]

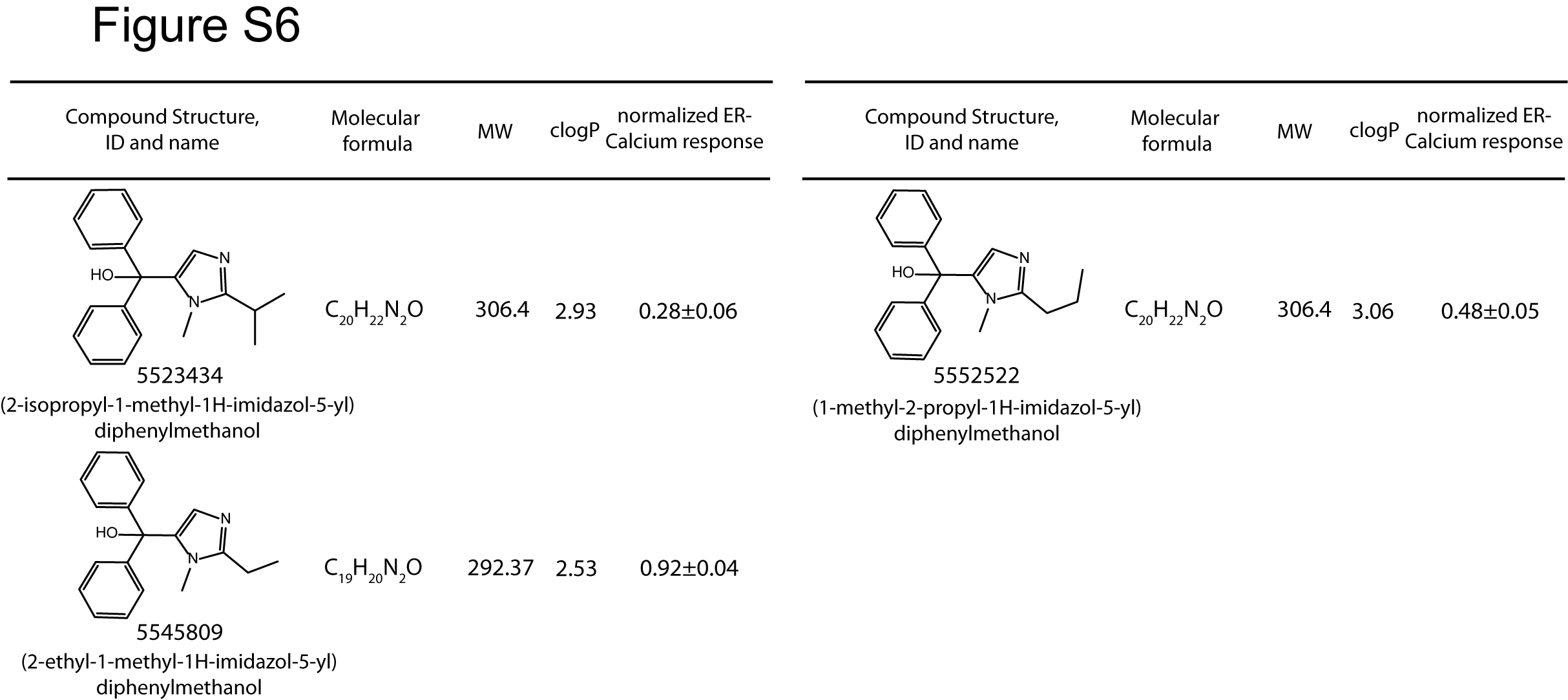

Supplement: Figure S6 — Imidazole lead structure. Shown are the 3 compounds belonging to the lead structure Imidazole. Their chemical structure, physical properties and mean normalized CCh-evoked calcium release peak response ± standard deviation are presented at 10 µM as a measure for their activity in the ER calcium release assay. (TIF) [file pone.0080645.s006.tif]

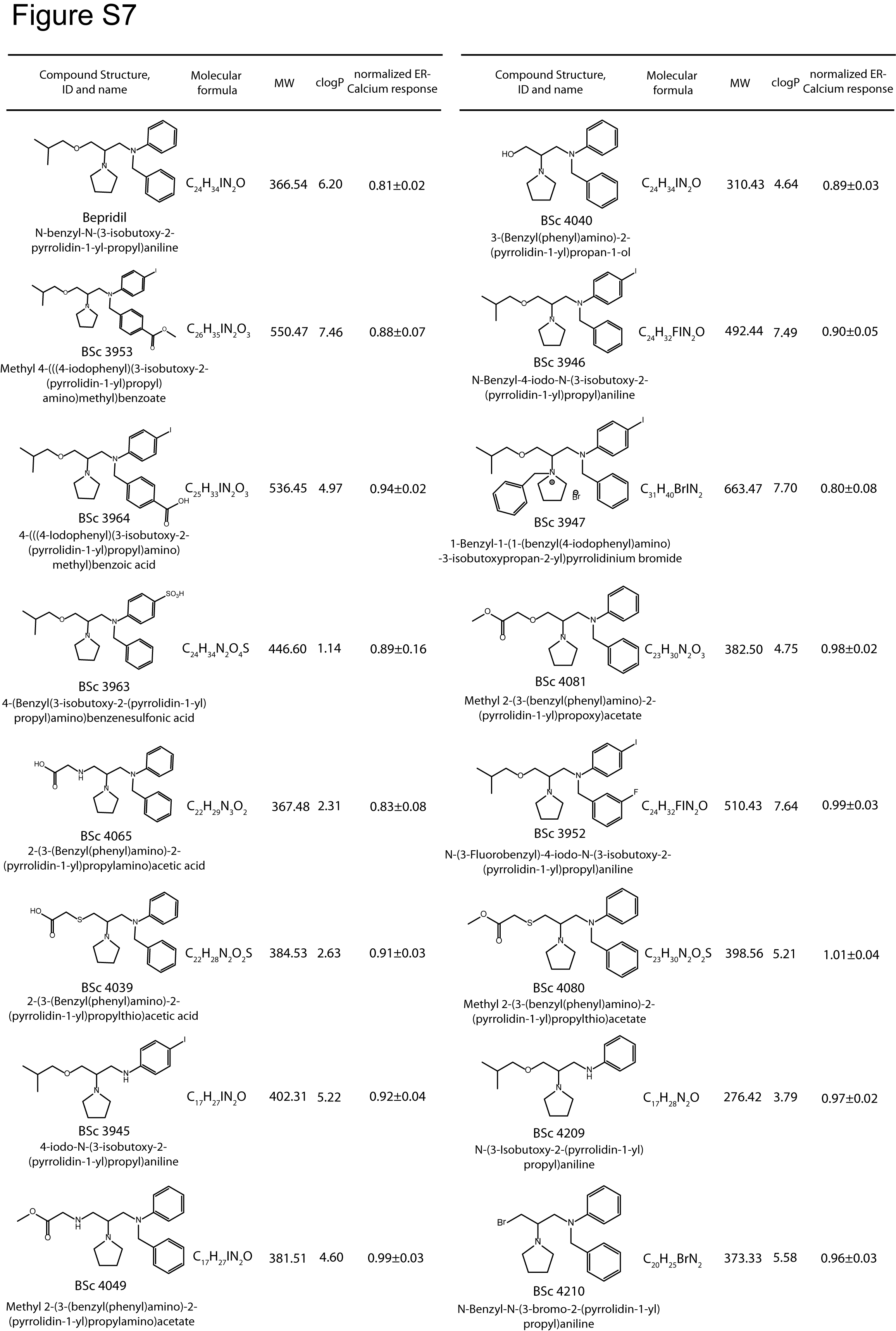

Supplement: Figure S7 — Bepridil lead structure. Shown are Bepridil and 15 synthesized derivatives, their chemical structure, physical properties and the mean normalized CCh-evoked calcium release peak response± standard deviation at 10 µM as a measure for their activity in the ER calcium release assay. (TIF) [file pone.0080645.s007.tif]

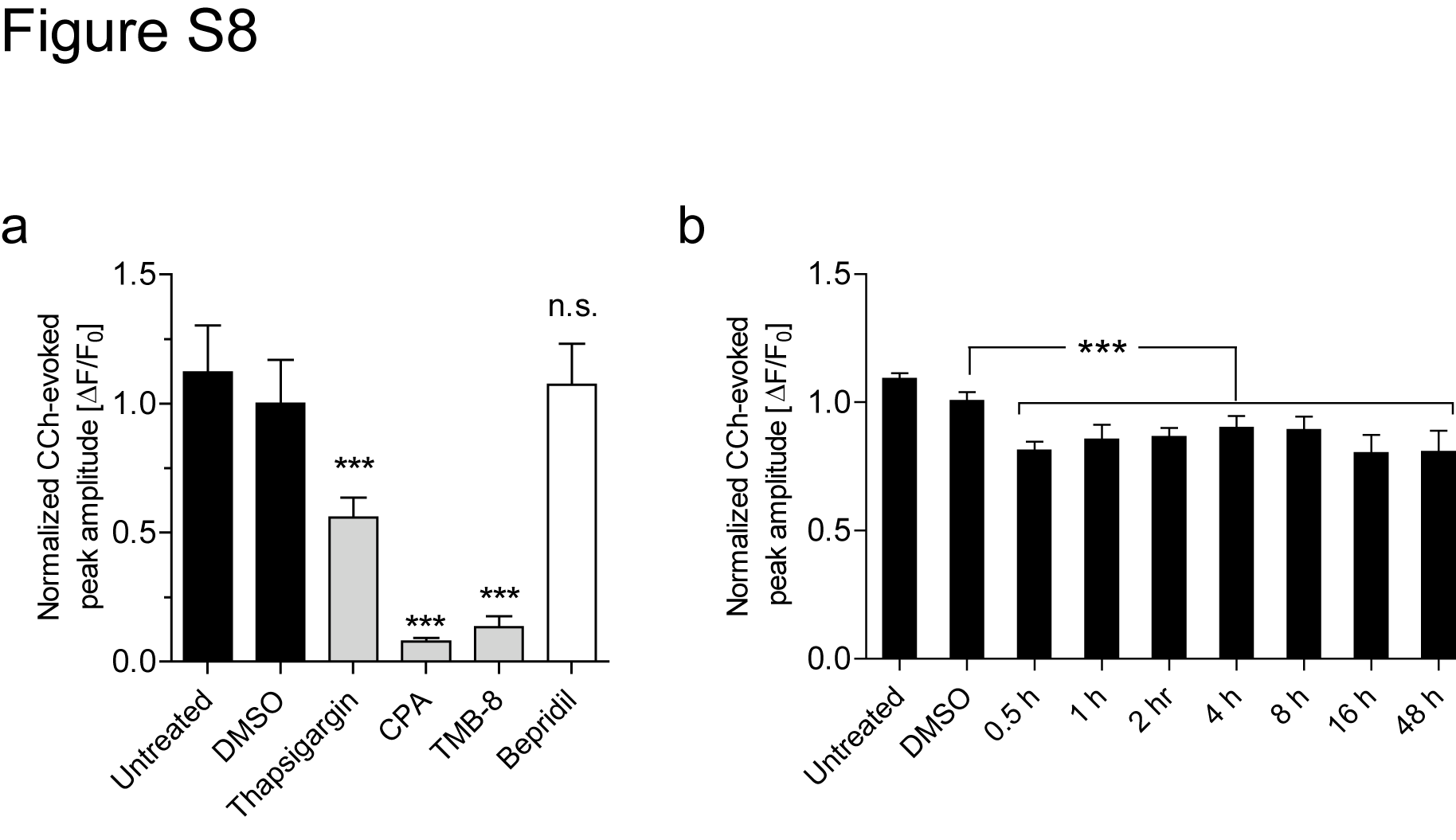

Supplement: Figure S8 — Characterization of the effect of Bepridil on the amplitude of CCh-evoked ER calcium release. (a) Bepridil (30 µM) does not alter the amplitude of CCh-evoked ER calcium release in wildtype PS1-expressing HEK293 cells. The peak response of DMSO-treated control is set to one. Thapsigargin (1 µM), CPA (20 µM) and TMB-8 (50 µM) were used as positive controls. (b) The time course of Bepridil (10 µM) incubation effect on the amplitude of normalized CCh-evoked ER calcium release in HEK293 cells expressing PS1-M146L. (n.s.: non-significant and *** P<0.001). (TIF) [file pone.0080645.s008.tif]

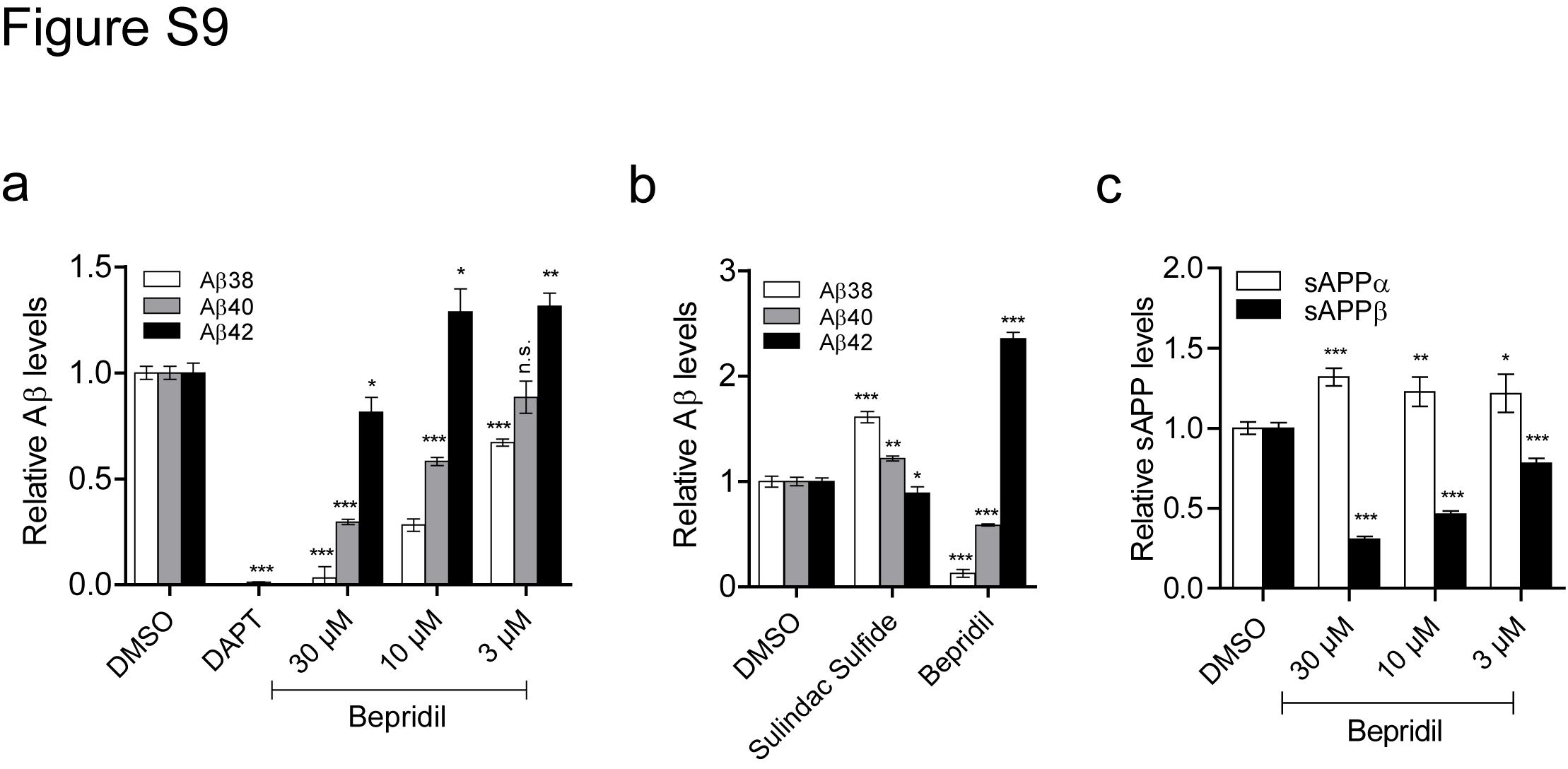

Supplement: Figure S9 — Effect of on Bepridil on APP processing. (a) Altered production of Aβ38, Aβ40 and Aβ42 after 16 h treatment of APP-overexpressing HEK293 cells with Bepridil. DAPT (10 µM), a γ-secretase inhibitor, was used as a positive control. (b) Altered production of Aβ38, Aβ40 and Aβ42 after 16 h treatment of C99-overexpressing HEK293 cells with Bepridil (30 µM). Sulindac sulfide (50 µM), a γ-secretase modulator, was used as a positive control. (c) Increased levels of sAPPα and decreased sAPPβ secreted fragments after 16 h treatment with Bepridil in APP-overexpressing HEK293 cells. (n.s.: non-significant; * P<0.05, ** P<0.01 and *** P<0.001). (TIF) [file pone.0080645.s009.tif]

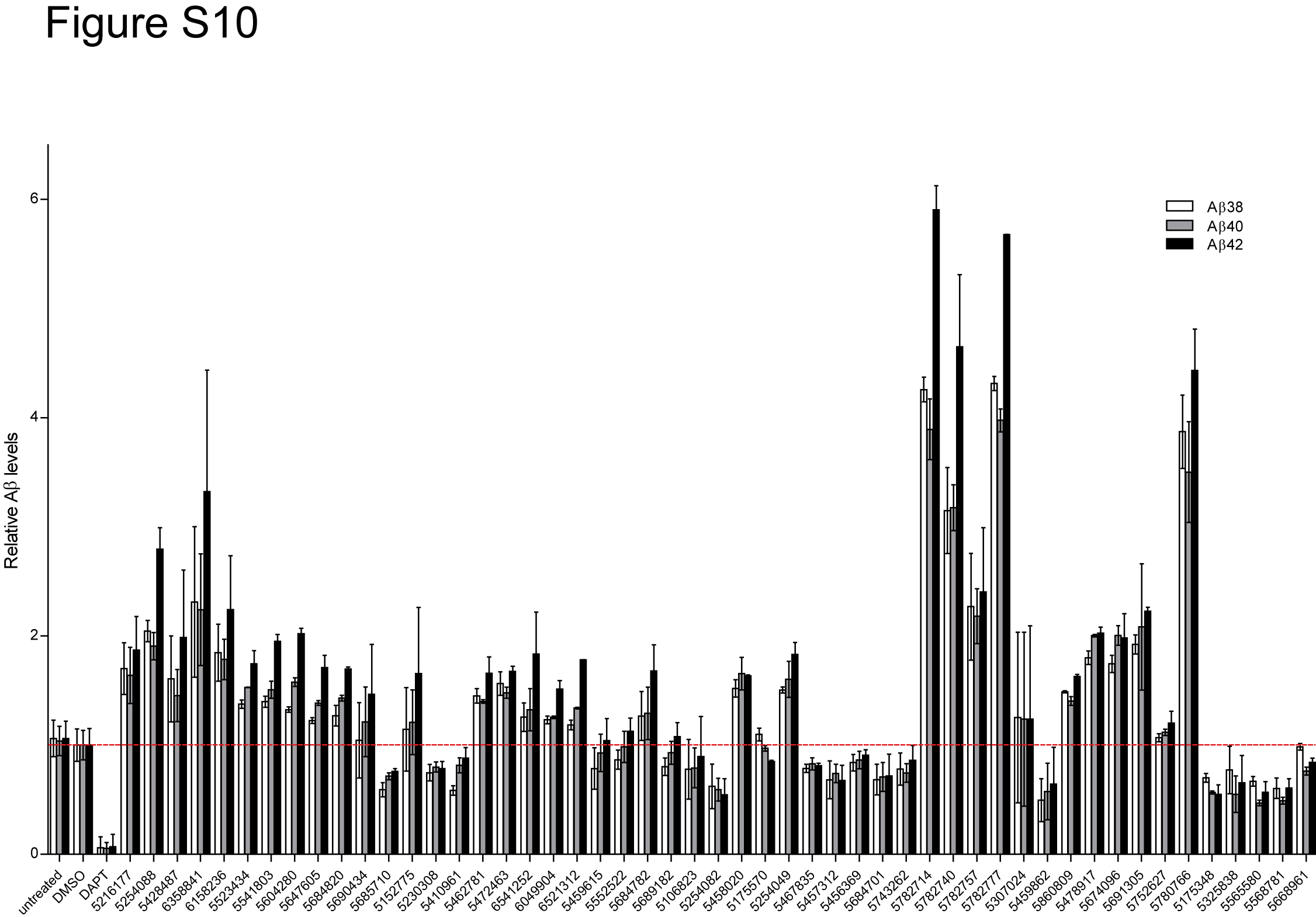

Supplement: Figure S10 — Effects of the active compounds from the calcium HTS on Aβ production. Altered production of Aβ38, Aβ40 and Aβ42 after 16 h treatment of HEK293 cells coexpressing APPsw and PS1-M146L with the active structures identified from the calcium HTS. DAPT (10 µM) was used as a γ-secretase inhibitor control. (TIF) [file pone.0080645.s010.tif]
